# 856. Postoperative Takedown Days as an Influence on Length of Stay

**DOI:** 10.1093/jbcr/irag033.326

**Published:** 2026-04-06

**Authors:** Justin Burleson, Scott W Mueller, Arek J Wiktor, Fred Endorf, Alexandra Halevi, Cameron Gibson

**Affiliations:** UCHealth Burn & Frostbite Center - Anschutz Medical Campus, Aurora, Colorado; UCHealth Burn & Frostbite Center - Anschutz Medical Campus, Aurora, Colorado; UCHealth Burn & Frostbite Center - Anschutz Medical Campus, Aurora, Colorado; University of Colorado School of Medicine, Aurora, Colorado; UCHealth Burn & Frostbite Center - Anschutz Medical Campus, Aurora, Colorado

## Abstract

**Introduction:**

A recent literature review revealed a lack of evidence regarding optimal timing for postoperative dressing removal following autograft surgery in burn patients. At our burn center, the decision for postoperative takedown (POTD) timing was left to individual provider preference—some preferring postoperative day 4, while others, day 5. All surgeons agreed to adopt a standardized POTD protocol on day 4.

**Methods:**

A retrospective study was conducted, comparing outcomes from one year prior to and one year after the implementation of standardized POTD on day 4. Patients were included only if they had undergone autografting as their sole surgical intervention during hospitalization (escharotomies excluded). Patients who underwent autologous dermal autografting were excluded as well. For the pre-implementation cohort, their surgical procedure to discharge had to exceed 4 days, while for the post-implementation cohort; it had to exceed 3 days.

**Results:**

The pre-implementation cohort included 54 patients; 67% were male, with a median age of 48 years, median total body surface area (TBSA) burned of 3.45%, and median LOS of 12 days. The post-implementation cohort included 67 patients; 68% were male, with a median age of 51 years, median TBSA burned of 3.75%, and median LOS of 11 days. In the pre-implementation group no patients experienced graft loss requiring a repeat procedure, and one patient in the post implementation group did.

Wilcoxon rank sum test was run to compare scores between POTD day 4 vs 5 groups. For LOS from surgery to discharge, the results indicated that this intervention trended towards a difference between the groups, but not statistically so, *p*=.1.

**Conclusions:**

Though a link between POTD on day 4 to decrease LOS was not shown in this review, given fiscal needs, capacity of units that specialize in burn care and an overall push to decrease LOS, a uniform approach to POTD may help decrease overall LOS without influencing graft take.

**Applicability of Research to Practice:**

Uniformity in POTD may be a way to help reduce the LOS for patients and help reduce costs. As overnight stays in the hospital approach $20 000 in total, economy of treatment becomes more pressing. These methods could easily be trialed at other burn centers and results shared to help find an optimal time for taking down autograft dressings postoperatively.

**Funding for the study:**

N/A.

